# Revealing biomass heterosis in the allodiploid x*Brassicoraphanus*, a hybrid between *Brassica rapa* and *Raphanus sativus*, through integrated transcriptome and metabolites analysis

**DOI:** 10.1186/s12870-020-02470-9

**Published:** 2020-06-03

**Authors:** Gibum Yi, Hosub Shin, Hye Rang Park, Jeong Eun Park, Jong Hwa Ahn, Sooyeon Lim, Jeong Gu Lee, Eun Jin Lee, Jin Hoe Huh

**Affiliations:** 1 Department of Plant Science, Seoul National University, Gwanak-gu, Seoul, 08826 South Korea; 2 Plant Genomics and Breeding Institute, Seoul National University, Seoul, 08826 South Korea; 3 Department of Central Area Crop Science, National Institute of Crop Science, RDA, Suwon, 16429 Republic of Korea; 4 Illumina Korea, Yeongdeungpo-gu, Seoul, 07325 South Korea; 5 Research Institute of Agriculture and Life Sciences, Seoul National University, Seoul, 08826 South Korea; 6 National Institute of Horticultural and Herbal Science, RDA, Wanju-gun, Jeollabuk-do 55365 South Korea

**Keywords:** Allodiploid, Biomass, Flowering time, Heterosis, Intergeneric hybrid, Sugar metabolism, Metabolome, Transcriptome

## Abstract

**Background:**

Heterosis is biologically important but the molecular basis of the phenomenon is poorly understood. We characterized intergeneric hybrids between *B. rapa* cv. Chiifu and *R. sativus* cv. WK10039 as an extreme example of heterosis. Taking advantage of clear heterosis phenotypes and the genetic distance between parents, we performed transcriptome and metabolite analysis to decipher the molecular basis of heterosis.

**Results:**

The heterosis was expressed as fresh weight in the field and as inflorescence stem length in the glass house. Flowering time, distributed as a normal segregating population, ranged from the early flowering of one parent to the late flowering of the other, in contrast to the homogeneous flowering time in a typical F1 population, indicating unstable allelic interactions. The transcriptome and metabolome both indicated that sugar metabolism was altered, suggesting that the change in metabolism was linked to the heterosis. Because alleles were not shared between the hybridized genomes, classic models only partly explain this heterosis, indicating that other mechanisms are involved.

**Conclusion:**

The differential expression of genes for primary and secondary metabolism, along with the altered metabolite profiles, suggests that heterosis could involve a change in balance between primary and secondary metabolism.

## Background

Heterosis, the tendency of hybrids to perform better than their parents, can be observed in phenotypes such as biomass, seed number, plant height, etc. Many of these hybrid phenotypes enhance yield and other agronomically important characteristics and therefore are exploited in a variety of breeding programs. Heterosis also occurs in many other organisms, including both plants and animals, suggesting that a fundamental mechanism underlies heterosis. However, although many explanations have been proposed, the mechanism of heterosis remains to be elucidated.

Several classical models for heterosis were based on allelic interactions. The dominance model explained heterosis as the sum of dominant alleles [1], whereas the overdominance model assumed that some heterozygous loci are more beneficial than homozygous loci [2], and the epistasis model emphasized interactions among loci [3]. More recently proposed molecular mechanisms for heterosis involve protein metabolism, energy use efficiency, and epigenetic factors [reviewed in [4–9]. One widely accepted concept is that the degree of heterosis is positively correlated with genetic distance. First suggested by East and Hayes [10], this concept originated based on observations with interspecific and intergeneric hybrids [11]. However, in limited ranges of genetic diversity, the opposite relationship has been reported [12, 13]. Thus, the search remains for a possible unifying model capable of explaining the mechanism of heterosis in both plants and animals.

The synthetic genus x*Brassicoraphanus* is an intergeneric hybrid of the cross between *Brassica rapa* L. and *Raphanus sativus* L. and is often referenced as an extreme example of heterosis because of the large genetic distance between parents [5, 11]. This hybrid has been repeatedly produced since Segeret first did so in 1826 [14]. Gravatt [15] and Kapchenko [16] introduced the gigantic plant known for its heterosis. Although the phenotype of its heterosis has distinct advantages, the production of the F1 hybrid from the parents *Brassica* and *Raphanus* is difficult because of hybrid incompatibility. Thus, systemic investigations have not been conducted on heterosis using x*Brassicoraphanus*, even though the effort to produce such hybrids has been continuous [17–19].

In this study, we characterized various aspects of heterosis in a synthetic allodiploid x*Brassicoraphanus*—the classic gigantic heterosis model—and investigated the biological mechanism associated with heterosis using transcriptome and metabolome analyses. Our results showed alterations in primary metabolism and suggest the importance of heterosis in evolution.

## Results

### F1 hybrids show hybrid vigor in shoot growth and biomass at the vegetative stage

We characterized the heterosis in the phenotypes of the F1 hybrid from the cross between *B. rapa* cv. Chiifu (CF) and *R. sativus* cv. WK10039 (WK) throughout development. The small, undeveloped F1 seeds rescued from 2-week-old siliques began to germinate at 3 days in vitro, and the seedlings were much smaller than normal parent seedlings. Considering seed development duration, before transplanting to soil, we grew the hybrid plants on MS medium for 20 more days than the *B. rapa* and *R. sativus* plants, which were germinated in vitro when transplanted to soil as parental controls. Because the F1 hybrid seeds were rescued from siliques that were not fully developed, it was not feasible to assess the hybrid phenotypes showing potential heterosis relative to the parents at early developmental stages, including traits such as cotyledon size, leaf size, and leaf initiating rate.

**Fig. 1:**
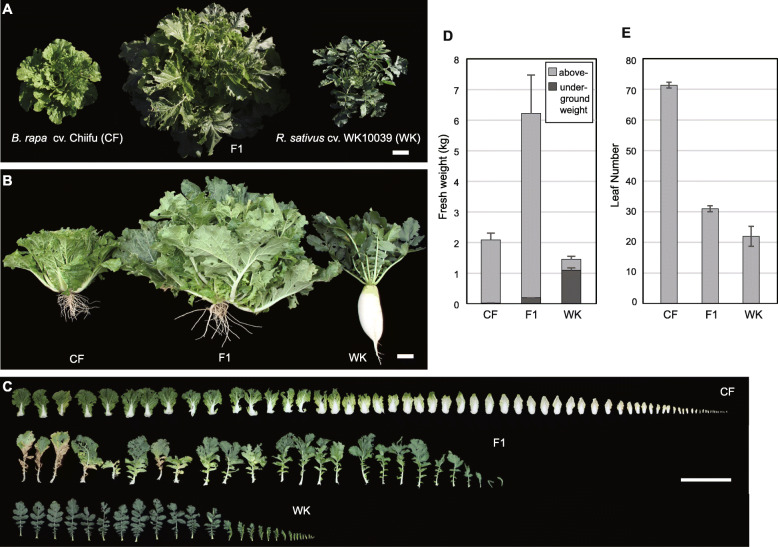
Heterosis of the F1 hybrid from the cross between *B. rapa* and *R. sativus* grown in the field. **a** Canopies of the F1 hybrid and the two parents. **b** Shoot and root phenotypes of the F1 hybrid and the two parents. Plants were photographed immediately after harvest in the field. The scale bar in **a**, also applicable to **b**, equals 10 cm. **c** A continuum of leaf shapes and the number of leaves for *B. rapa*, *R. sativus*, and the F1 hybrid. The scale bar equals 30 cm. **d** Total fresh weights of plants with root weights (*n* = 3). Bars indicate average values ± standard deviations. **e** Average number of leaves ± standard deviations (*n* = 3)

The hybrids and parents were transplanted in the field in early September in 2016, and the vigor phenotype of the F1 hybrid became noticeable after 1 month (Fig. 1). We measured plant fresh weight 2 months later, at the end of the growing season. The F1 hybrids were 3- and 4-fold heavier (6.21 ± 1.29 kg) than the maternal and paternal parents (2.02 ± 0.28 and 1.36 ± 0.13 kg), respectively (Table 1; Fig. 1a, b, and d). The color of the F1 leaves was intermediate between the darker green of WK plants and the lighter green of CF plants (Fig. 1a). The number of leaves (31.0 ± 1.0) of the hybrids was intermediate compared with that of the maternal (71.3 ± 0.9) and paternal (22.0 ± 3.3) parents but less than middle parent value (MPV, 46.7). The canopy of the hybrids (114.3 ± 5.1 cm in diameter) was 2.7- and 2.2-fold larger than that of the maternal (41.6 ± 1.5 cm) and paternal (51.4 ± 1.5 cm) parents, respectively (Table 1; Fig. 1a).

**Table 1:** Phenotypes of the F1 hybrids and their parents

Phenotype		*B. rapa*	F1	*R. sativus*	MPV*
Vegetative	Biomass (kg)	2.02 ± 0.28 (*n* = 4)	6.21 ± 1.29 (*n* = 3)	1.36 ± 0.13 (*n* = 5)	1.69
	Canopy diameter (cm)	41.6 ± 1.5 (*n* = 3)	114.3 ± 5.1 (*n* = 3)	51.4 ± 1.5 (*n* = 3)	46.5
	Leaf number (ea)	71.3 ± 0.9 (*n* = 3)	31.0 ± 1.0 (*n* = 3)	22.0 ± 3.3 (*n* = 3)	46.7
Reproductive	Inflorescence stem length (cm)	62.38 ± 2.53 (*n* = 4)	157.78 ± 26.73 (*n* = 24)	103.67 ± 17.21 (*n* = 3)	83.03
	Inflorescence node number (ea)	103.25 ± 8.98 (*n* = 4)	136.03 ± 19.60 (*n* = 24)	54.33 ± 7.41 (*n* = 3)	78.79
	Internode length (cm)	0.61 ± 0.05 (*n* = 4)	1.17 ± 0.20 (*n* = 24)	1.90 ± 0.12 (*n* = 3)	1.26
	Flowering time (days after planting)	349.3 ± 4.1 (*n* = 3)	312.0 ± 28.1 (*n* = 23)	272.7 ± 4.5 (*n* = 3)	311

*MPV: middle parent value

CF leaves were characterized by a large mid vein and round shape, whereas WK plants had lyrate leaves with a large terminal lobe and small rounded lobes toward the base (Figs. 1c and S1). The F1 hybrid plants produced more sinuate leaves with less lobes, the form of which was intermediate to those of the parents (Fig. S1). Notably, the F1 hybrids produced larger leaves than the parents (Fig. 1c). Leaf and pollen shapes similarly showed a distribution of phenotypes (Figs. S1 and S2). Because of the approach of winter, we could not observe the phenotypes of the reproductive stages in the field.

In the field, the root growth of WK was much greater than that of CF and F1 plants, and the proportion of underground fresh weight to the total fresh weight was 0.8% for CF, 3% for F1, and 75% for WK. For WK plants, we considered the whole root as underground tissue, even though a portion of the root was aboveground and tuberized from the hypocotyl [20]. The CF plants produced numerous secondary roots with one primary root, the WK plants had a primary tab root with few secondary roots, and the F1 plants had many secondary roots and the growth of a primary root that were similar to those of CF plants (Fig. 1b).

### Heterosis in inflorescence growth of the hybrids at the reproductive stage

We also observed heterosis in inflorescence stem length and node number in glass house-grown F1 plants (Table 1). The F1 hybrids had an indeterminate flowering pattern with elongated inflorescence stems compared to the parents, whereas the number and length of inflorescence stem that proliferated were limited in CF and WK plants. For F1 plants grown in pots in the glass house, the difference in growth vigor was not as distinct as that in the field. This result could be explained, in part, because the older leaves often perished in pots grown under glass house conditions. The difference in growth vigor that we observed between plants grown in the field and those in pots suggested that the hybrids needed additional nutrients for growth.

**Fig. 2:**
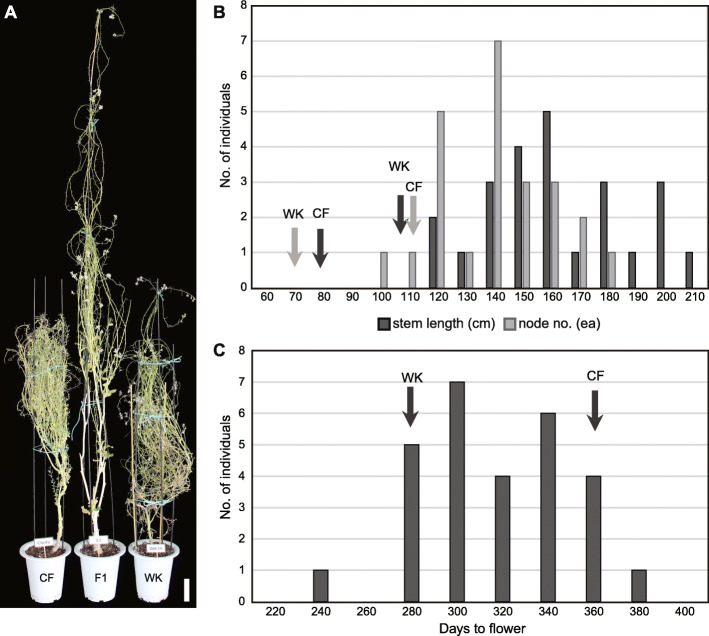
Growth vigor is consistent despite heterogeneous phenotypes in F1 hybrids. The hybrid phenotypes were observed in 28 F1 plants and parental controls grown in a glass house. **a** Heterosis in plant height of the F1 hybrid compared to that of its parents. The scale bar equals 10 cm. **b** Inflorescence stem length and number of nodes, which were measured for three stems for each plant and distributed by their average values. Arrows indicate values for parents. **c** Flowering time. The day after planting when bolting was visible determined flowering time. Arrows indicate values for parents

Of the 28 F1 hybrids in the glass house (Table S1), all had longer inflorescence stem than the parents, with a fairly normal distribution (Table 1; Fig. 2a, b). The average node length (1.17 ± 0.20 cm) of the hybrids was between those of the parents (0.61 ± 0.05 and 1.90 ± 0.12 cm) and very close to MPV (1.26 cm), suggesting that an increase in plant height was due to producing more flowers with a longer maintenance period of floral meristem (Table 1). Both parents were self-incompatible; therefore, the flowers all failed to set seed, with the same being observed for the sterile F1 hybrid. Thus, fertility could not explain the extended period of floral meristem persistence in the F1 hybrid. Flowering time of the F1 hybrids (312.0 ± 28.1) was close to MPV (311), and was normally distributed from the time of the early flowering WK (272.7 ± 4.5) to that of the late flowering CF (349.3 ± 4.1) (Table 1; Fig. 2c). The allodiploidy of the F1 hybrids were confirmed by counting chromosome numbers from the pollen mother cells in meiosis (Fig. S3). Notably, the flowering time of the F1 hybrids was asynchronous, which suggested that flowering of the F1 hybrid was under stochastic regulation that might involve unstable interactions between the parental alleles upon intergeneric hybridization.

### F1 hybrids show alteration in metabolite profiles

To understand the cause of heterotic phenotypes observed in the F1 plants, we analyzed 33 metabolites from the leaves, including 10 sugars, 13 sugar acids, and one fatty acid, among others. The CF plants had more 3C polysaccharides, such as glycerol and D-lactic acid, than either WK or F1 (Fig. S4). The WK plants had more monosaccharides than CF plants, including mannose, fructose, glucose, and inositol, as well as more 5C sugar acids, such as ketoglutaric acid, ribonic acid, and arabinonic acid (Fig. S4). However, the F1 plants had more 4C polysaccharides, such as maleic acid, malic acid, fumaric acid, and L-threonic acid, than CF and WK plants. Furthermore, most of the F1 hybrids had more ribofuranose and furanone and less fructose and floridoside than either parent. We detected D-turanose in WK but not in CF plants, whereas approximately one-half of the F1 plants contained D-turanose. These data indicate that primary sugar metabolism is substantially altered in x*Brassicoraphanus* F1 plants compared to the parents.

**Fig. 3:**
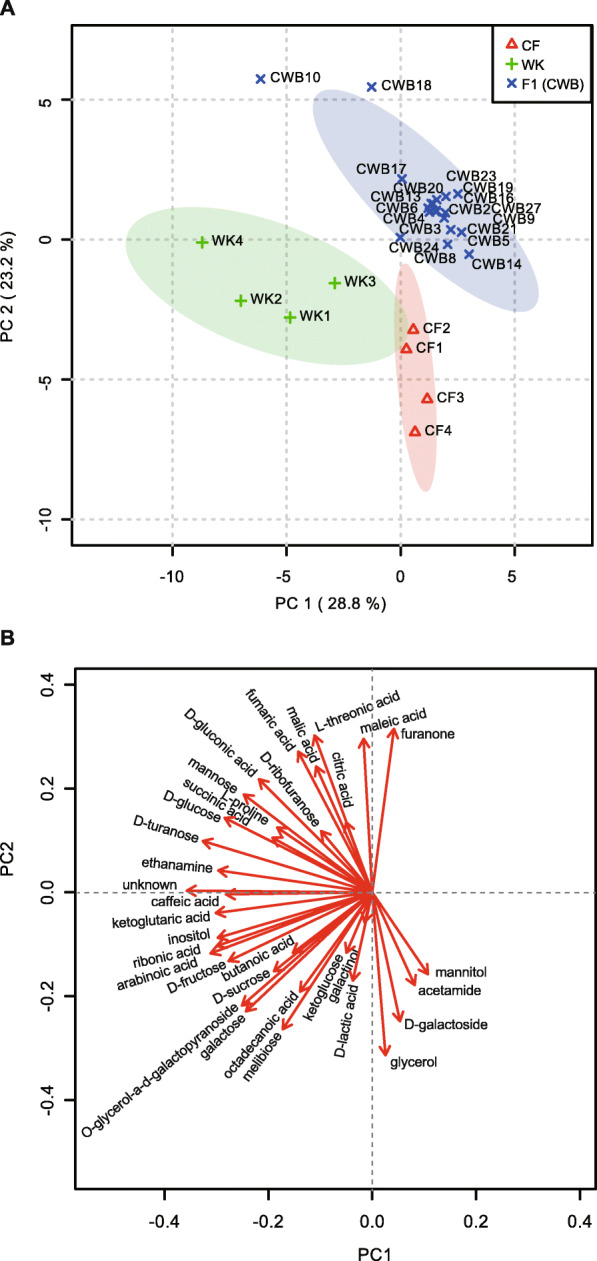
Principal component analysis of the F1 hybrid and its parents based on metabolite profiles. Loading plot **a** and scoring plot **b** of 34 metabolites

Principal component (PC) analysis revealed that F1 plants and the parents had distinct metabolite profiles (Fig. 3). PC1 and PC2 explained 52% of all variance. The CF and WK samples were located diagonally in the fourth and third quadrants, respectively (Fig. 3a). All the F1 samples (referred to as CWB hereafter) except for CWB10 and CWB18, surrounded the center of the plot or were placed nearby, where the 95% of confidence limits of CF and WK plants overlapped. Such distribution showed that, although CF and WK samples were variable within samples for specific metabolites, the metabolic profiles of the F1 plants were generally intermediate to the parents (Fig. 3a). These results suggest that altered sugar metabolism may contribute to the heterotic phenotypes in x*Brassicoraphanus* F1 plants at both vegetative and reproductive stages even though there is no dramatic increase or decrease in the amount of a specific metabolite(s).

### The transcriptome of the F1 hybrids showed alterations in primary and secondary metabolism

To assess the relationship between the heterotic phenotypes and a change in metabolite profile in x*Brassicoraphanus* F1 plants, we prepared the RNA libraries from three biological replicates of *B. rapa* (CF) and *R. sativus* (WK) and 20 individuals of the F1 hybrid and performed transcriptome analysis (Table S2). After filtering the raw reads, the sequence reads from CF, WK, and F1 plants were mapped onto the reference genomes of *B. rapa* (A genome [21];) and *R. sativus* (R genome [22];) and the hypothetical genome of x*Brassicoraphanus* F1, where both A and R genomes were integrated together. The expression levels of several genes in CF, WK, and F1 hybrids were validated with qPCR and showing similar patterns with FPKM values (Figs. S5 and S6).

**Fig. 4:**
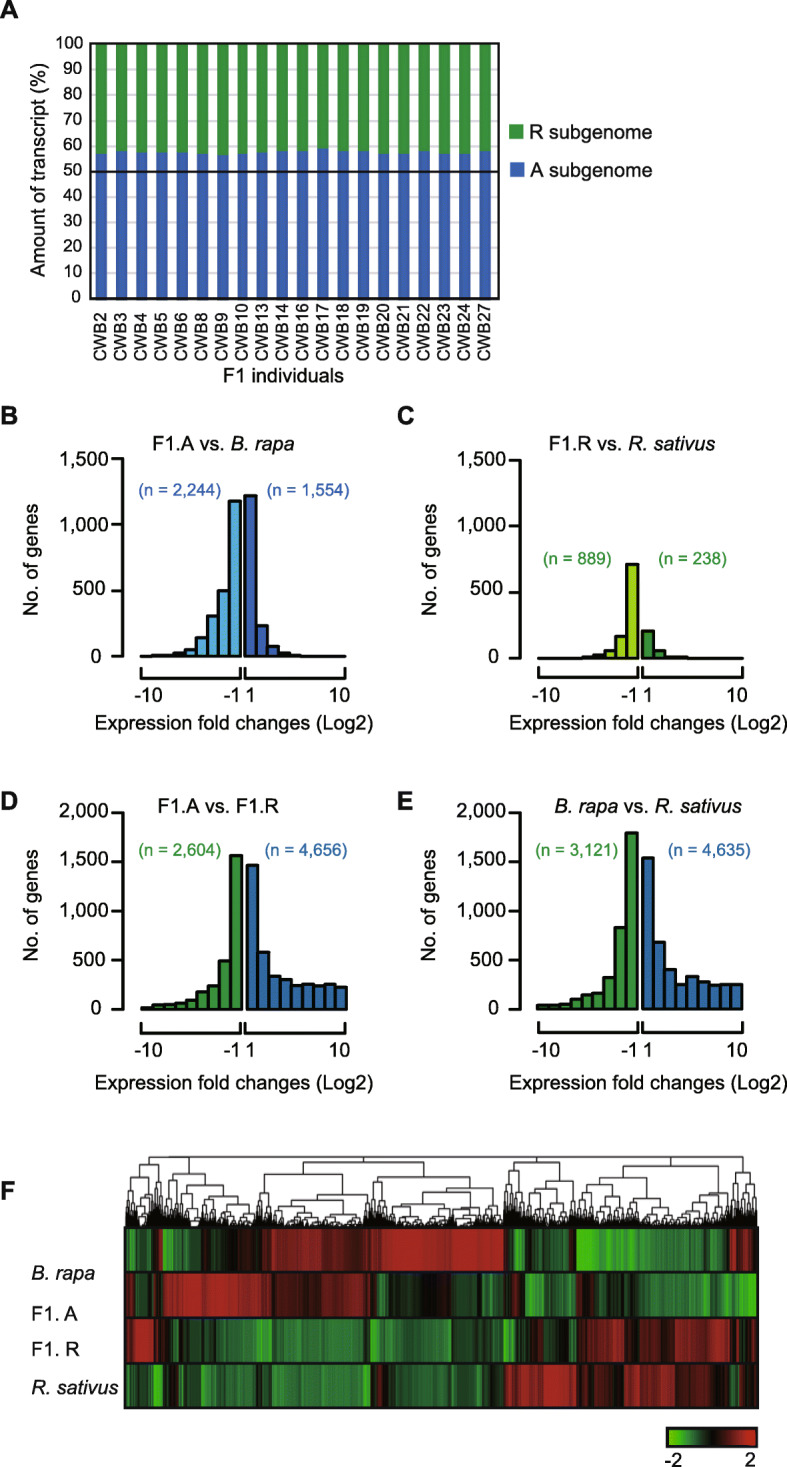
Transcriptome analysis of the F1 hybrids and their parents. **a** Parental proportions of transcripts in the F1 hybrids. **b-e** Bar graphs show the number of differentially expressed genes. **f** Hierarchical cluster of gene expression of Br-F1.A-F1.R-Rs orthologous genes. A total of 10,005 genes differentially expressed in at least one of the four pairs are presented. The color key represents the normalized z-score value

To explore the changes in gene expression upon hybridization, we counted the mapped reads for the 21,538 orthologous gene pairs from F1 plants into A and R subgenomes and compared the expression of genes from each subgenome. Of all transcripts in the F1 hybrids, over half of the reads (55%) were mapped to the genes from A genome, while the remaining 45% were mapped to the R genome derived genes (Fig. 4a).

To characterize the expression differences between the A and R genomes, we performed pairwise comparisons of the expression levels of 21,538 orthologous genes. In the F1 hybrids, 1554 genes were up-regulated and 2244 were down-regulated for the A subgenome, whereas 238 genes were up-regulated and 889 were down-regulated for the R subgenome (Fig. 4b, c). Notably, a significant fraction (~ 77%) of the differentially expressed genes (DEGs) originated from the maternal A genome, suggesting that a parental bias was maintained in the F1 hybrids. When comparing the A and R genomes, we detected 4635 A-biased and 3121 R-biased genes in the parents before hybridization, whereas we detected 4656 A-biased and 2604 R-biased genes in the F1 hybrid after hybridization, showing that the number of genes with biased expression was fairly conserved upon hybridization (Fig. 4d, e). According to the altered expression of the DEGs observed in the F1 plants, all possible modes, including additive, dominant, underdominant, and overdominant, existed in the F1 hybrids (Fig. 4f).

**Fig. 5:**
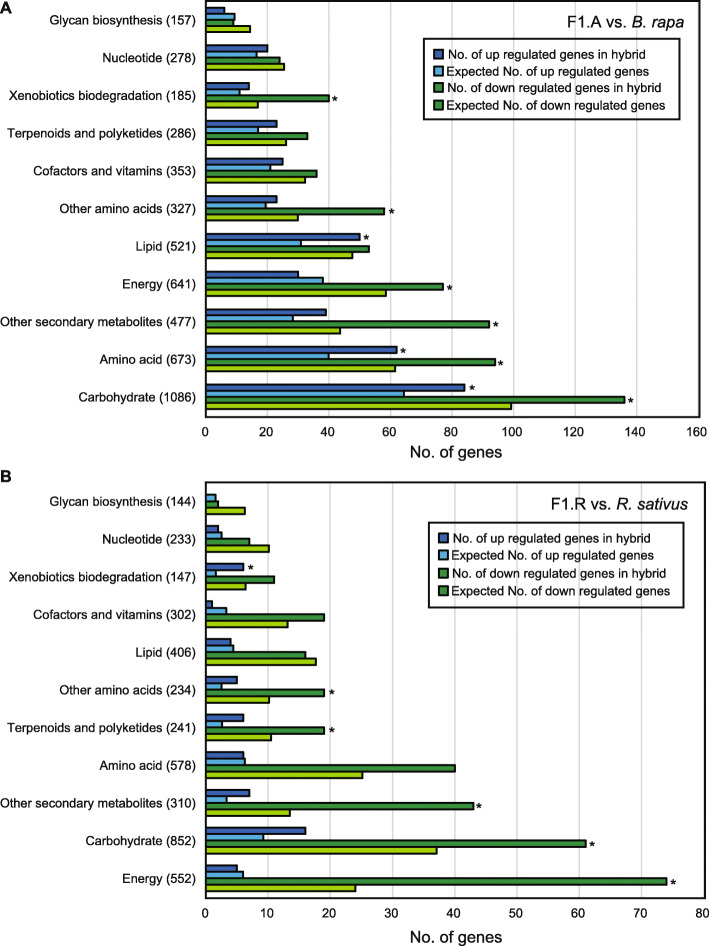
Enrichment of DEGs for various metabolisms by KEGG analysis. DEGs for the F1 versus maternal and paternal parents are separated in **a** and **b**, respectively. The number of assigned genes in each pathway is shown in parentheses. Blue and green bars show the number of up- and down-regulated genes in the hybrid, respectively. Enrichment tests were performed with Fisher’s exact test, and significant values are represented with an asterisk (*p* < 0.05)

To evaluate the function of DEGs, 6028 and 1539 of the DEGs between F1 and the parents were annotated with gene ontology (GO) terms and the GO enrichment tests with *p* < 0.0005 in each biological process category (Tables 2 and 3). For the up-regulated genes in the F1 hybrids, GO terms related to catabolic processes were significantly enriched in F1, which were commonly detected between the A and R genomes. The processes included the ‘cellular carbohydrate catabolic process’ (GO:0044275), ‘glucan catabolic process’ (GO:00092521), ‘cellular polysaccharide catabolic process’ (GO:0044247), ‘glucan metabolic process’ (GO:0006073), ‘starchy catabolic process’ (GO:0005983), and ‘starch metabolic process’ (GO:0005977). Thus, vigorous growth of the F1 hybrids might be related to the changes in catabolic activities in both genomes. For the down-regulated genes, the response to stress was overrepresented in both A and R subgenomes of x*Brassicoraphanus* (Fig. 5), whereas photosynthesis-related GO terms were overrepresented only in the down-regulated genes of the R genome (Table 3; Fig. 5).

**Table 2:** The top 15 most represented GO terms of up- and down-regulated genes for the A genome of F1 vs. *B*. *rapa* in the biological process category

	GO	Term	Anno-tated	Sig-nificant	Expe-cted	Fisher	FDR	Level
UP	GO:0044275	Cellular carbohydrate catabolic process	92	25	6.75	6.30E-09	2.89E-05	5
	GO:0009251	Glucan catabolic process	65	20	4.77	2.00E-08	4.59E-05	7
	GO:0044247	Cellular polysaccharide catabolic process	67	20	4.91	3.60E-08	5.50E-05	6
	GO:0003002	Regionalization	247	43	18.11	9.10E-08	9.17E-05	5
	GO:0007389	Pattern specification process	292	48	21.41	1.00E-07	9.17E-05	4
	GO:0048513	Organ development	1389	152	101.84	2.50E-07	0.000191	4
	GO:0009888	Tissue development	727	91	53.3	3.10E-07	0.000203	4
	GO:0010065	Primary meristem tissue development	16	9	1.17	4.30E-07	0.000246	5
	GO:0016553	Base conversion or substitution editing	13	8	0.95	7.50E-07	0.000335	7
	GO:0006073	Cellular glucan metabolic process	313	48	22.95	8.60E-07	0.000335	6
	GO:0044042	Glucan metabolic process	313	48	22.95	8.60E-07	0.000335	6
	GO:0048731	System development	2528	246	185.34	9.00E-07	0.000335	4
	GO:0048508	Embryonic meristem development	48	15	3.52	9.50E-07	0.000335	4
	GO:0008283	Cell proliferation	176	32	12.9	1.50E-06	0.000491	3
	GO:0007169	Transmembrane receptor protein tyrosine kinase signaling pathway	86	20	6.31	3.00E-06	0.000917	7
DOWN	GO:0006950	Response to stress	4318	714	457.6	< 1E-30	< 1E-30	3
	GO:1901700	Response to oxygen-containing compound	2498	473	264.72	< 1E-30	< 1E-30	4
	GO:0010200	Response to chitin	213	98	22.57	< 1E-30	< 1E-30	5
	GO:0006952	Defense response	1603	336	169.88	< 1E-30	< 1E-30	4
	GO:0010243	Response to organo-nitrogen compound	250	103	26.49	< 1E-30	< 1E-30	4
	GO:0050896	Response to stimulus	7441	1045	788.56	< 1E-30	< 1E-30	2
	GO:0042221	Response to chemical	4025	637	426.55	< 1E-30	< 1E-30	3
	GO:1901698	Response to nitrogen compound	351	118	37.2	< 1E-30	< 1E-30	4
	GO:0001101	Response to acid chemical	1916	358	203.05	1.50E-29	6.87E-26	4
	GO:0009607	Response to biotic stimulus	1248	252	132.26	1.90E-25	4.35E-22	3
	GO:0043207	Response to external biotic stimulus	1238	248	131.2	1.60E-24	1.83E-21	4
	GO:0051707	Response to other organism	1238	248	131.2	1.60E-24	1.83E-21	3
	GO:0010033	Response to organic substance	2991	481	316.97	2.40E-24	2.20E-21	4
	GO:0009751	Response to salicylic acid	358	103	37.94	5.70E-22	4.35E-19	5
	GO:0006979	Response to oxidative stress	644	146	68.25	1.40E-19	9.16E-17	4

**Table 3:** The top 15 most represented GO terms of up- and down-regulated genes for the R genome of F1 vs. *R*. *sativus* in the biological process category

	GO	Term	Anno-tated	Sig-nifi-cant	Expect-ed	Fisher	FDR	Level
UP	GO:0080027	Response to herbivore	27	7	0.35	4.10E-08	0.000192	4
	GO:0005983	Starch catabolic process	29	7	0.38	7.10E-08	0.000192	7
	GO:0005982	Starch metabolic process	94	10	1.22	4.10E-07	0.00074	7
	GO:0009251	Glucan catabolic process	80	9	1.04	9.70E-07	0.001313	7
	GO:0044247	Cellular polysaccharide catabolic process	87	9	1.13	2.00E-06	0.002166	6
	GO:0010597	Green leaf volatile biosynthetic process	4	3	0.05	8.60E-06	0.006653	7
	GO:0019372	Lipoxygenase pathway	4	3	0.05	8.60E-06	0.006653	6
	GO:0005977	Glycogen metabolic process	25	5	0.32	1.50E-05	0.008664	5
	GO:0006112	Energy reserve metabolic process	25	5	0.32	1.50E-05	0.008664	5
	GO:0044275	Cellular carbohydrate catabolic process	112	9	1.45	1.60E-05	0.008664	5
	GO:0006690	Icosanoid metabolic process	8	3	0.1	0.00012	0.05415	5
	GO:1901568	Fatty acid derivative metabolic process	8	3	0.1	0.00012	0.05415	4
	GO:0009607	Response to biotic stimulus	1752	41	22.74	0.00019	0.07581	3
	GO:0043207	Response to external biotic stimulus	1704	40	22.12	0.00021	0.07581	4
	GO:0051707	Response to other organism	1704	40	22.12	0.00021	0.07581	3
DOWN	GO:0015979	Photosynthesis	366	67	13.44	5.70E-28	3.09E-24	4
	GO:0009416	Response to light stimulus	1211	107	44.46	2.70E-17	7.31E-14	5
	GO:0019684	Photosynthesis, light reaction	196	37	7.2	1.90E-16	3.43E-13	5
	GO:0009314	Response to radiation	1257	107	46.15	3.60E-16	4.87E-13	4
	GO:0009628	Response to abiotic stimulus	3421	213	125.6	1.60E-15	1.73E-12	3
	GO:0006091	Generation of precursor metabolites and energy	524	59	19.24	2.30E-14	2.08E-11	4
	GO:0055114	Oxidation-reduction process	2625	171	96.37	4.40E-14	3.40E-11	4
	GO:0044711	Single-organism biosynthetic process	2955	186	108.49	6.40E-14	4.15E-11	4
	GO:0050896	Response to stimulus	9671	465	355.06	6.90E-14	4.15E-11	2
	GO:0042221	Response to chemical	4843	267	177.81	4.00E-13	2.17E-10	3
	GO:0044550	Secondary metabolite biosynthetic process	489	54	17.95	6.50E-13	3.20E-10	5
	GO:0019748	Secondary metabolic process	692	66	25.41	1.60E-12	7.22E-10	4
	GO:0044710	Single-organism metabolic process	7483	372	274.73	2.90E-12	1.21E-09	3
	GO:0009765	Photosynthesis, light harvesting	71	19	2.61	6.40E-12	2.48E-09	5
	GO:0043436	Oxoacid metabolic process	2061	136	75.67	1.20E-11	4.33E-09	5

### Combined analyses of metabolites and transcriptomes suggest an important role for starch metabolism in heterosis

**Fig. 6:**
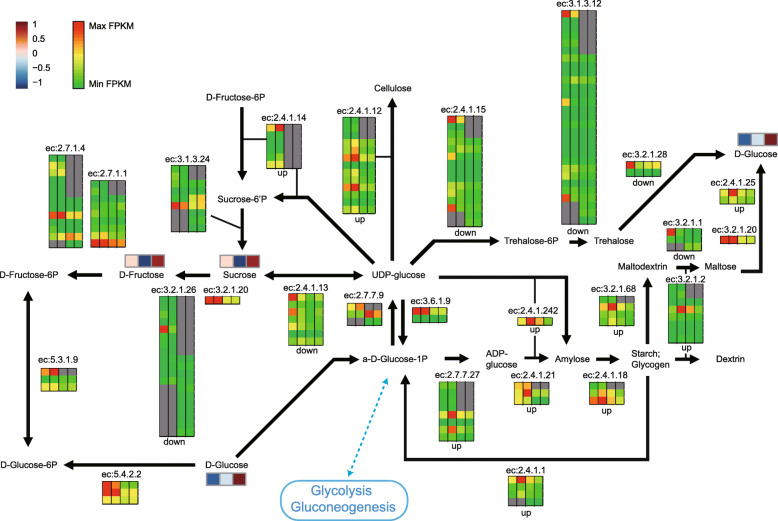
Alterations in sucrose and starch metabolism of the F1 hybrids. The expression levels of the corresponding genes for each enzyme are color-scaled in which fragments per kilobase million (FPKM) values were divided by the maximum FPKM among the same enzyme-encoded genes. The four columns from left to right indicate orthologs from CF, A subgenome of F1, R subgenome of F1, and WK, respectively. Gray boxes indicate that there were no corresponding genes. Each enzyme was designated ‘up’ or ‘down’ when there were differentially expressed genes. The amounts of metabolites were autoscaled and are indicated in the color scale. The three boxes from left to right indicate the amount of metabolites for CF, F1, and WK, respectively

To confirm the shift in metabolic activities in the F1 hybrid, we performed KEGG (Kyoto Encyclopedia of Genes and Genomes) enrichment analysis and found that various metabolic pathways were enriched, including the ‘starch and sucrose metabolism pathway’, ‘carbon fixation in photosynthetic organisms,’ and ‘glycolysis/gluconeogenesis’ (Figs. 6 and S7). In the ‘starch and sucrose metabolism pathway,’ the major expressed genes up-regulated upon hybridization were associated with glucose-1-phosphate adenylyltransferase (ec.2.7.7.27), ADP-glucose synthase (ec.2.4.1.21), NDP-glucose-starch glucosyltransferase (ec.2.4.1.242), 1,4-alpha-glucan branching enzyme (ec.2.4.1.18), and starch phosphorylase (ec.2.4.1.1), all of which are involved in starch biosynthesis from glucose. The maternal copies of those genes were up-regulated, whereas the paternal copies showed conservation of the level of expression (Fig. 6). Starch phosphorylase (ec.2.4.1.1), which catalyzes the inverse metabolism, was also up-regulated, suggesting more sugar and starch were produced and utilized in the F1 hybrids than in the parents. Other enzymes, including sucrose-phosphate synthase (ec.2.4.1.14), sucrose synthase (ec.2.4.1.13), β-amylase (ec.3.2.1.2), and 4-α-glucanotransferase (ec.2.4.1.25), were differentially regulated, suggesting that the activity of the primary metabolic pathway was substantially altered in the F1 hybrids (Fig. 6). The metabolites that were presumably regulated by these pathways were also altered in F1 hybrids, suggesting that these transcriptome and metabolome changes might result in physiological changes leading to heterosis observed in the F1 plants. A decrease in the amount of sucrose and the down-regulation of sucrose biosynthesis were consistent, which suggests the possibility that more starch may accumulate in the F1 hybrids, eventually expressed as growth vigor.

The phenotypes, inflorescence stem length, node number, and flowering time of F1 hybrids were applied for the correlation analysis with metabolite profiles and gene expression (Fig. S8). Genes having positive or negative correlation (|Pearson’s coefficient| > 0.6) with the phenotype and metabolite contents were also selected and their enriched GO terms were investigated (Tables S3 and S4). For example, genes showing correlation with flowering time were enriched in GO terms related with cellulose biosynthesis in addition cellulose component such as D-fructose and D-glucose showed strong correlation (Pearson’s coefficient > 0.5) with flowering time phenotype. In addition, amount of D-fructose has correlation with genes enriched in primary cell wall biogenesis showing both metabolome and transcriptome represent correlations with the phenotype. Correlations between cell wall biogenesis and flowering time have been suggested in different studies [23, 24]. For heterosis phenotype, inflorescence stem length, D-gluconic acid and succinic acid showed the highest correlation with Pearson’s coefficient > 0.5 and mannose and D-fructose also showed positive correlations (Pearson’s coefficient > 0.4). For node number, only O-glycerol-a-d-galactopyranoside showed correlation with Pearson’s coefficient > 0.4. Genes showing expression correlation with the heterosis phenotype and those metabolites were enriched in GO terms related with fatty acid, oxoacid, organic acid metabolism.

## Discussion

### Growth vigor is consistent despite heterogeneous phenotypes in F1 hybrids

We assessed heterotic phenotypes of intergeneric hybrids x*Brassicoraphanus* from a cross between *B. rapa* and *R. sativus*, a classic extreme example of heterosis [5, 11]. The growth vigor of F1 hybrids in the field (Fig. 1) was reminiscent of the gigantic x*Brassicoraphanus* generated from a cross between *R. sativus* and *B. oleracea* [15]. The F1 plants showed variable phenotypes, i.e., flowering time and leaf shape (Figs. 2 and S1), which might result from the instability of the hybridized genome and transcriptome, described as genomic and transcriptomic shocks, respectively [25, 26]. However, despite such phenotypic variations, heterosis in biomass and plant height was consistently expressed in all F1 individuals. Karpechenko [16] also observed phenotypic variations in leaf shape and flowering time in F1 hybrids, where growth vigor in height or in the canopy area was apparent in 83 plants of 123 F1 hybrids obtained from a cross between *R. sativus* and *B. oleracea*. In those populations, dwarfism was also reported, although it was not observed in this study. Direct comparisons of growth differences between F1 hybrids and the parent plants are often hampered by different flowering time, particularly in the Brassicaceae, including the model plant Arabidopsis [27]. In this study, heterosis did not appear to be directly related to flowering time; whenever flowering occurred, the inflorescence stem length of F1 hybrids was always greater than that of either parent regardless of the time of onset of flowering (Fig. 2).

Heterosis is defined as any superior phenotype in an F1 hybrid compared to the parents. In F1 hybrids, heterosis occasionally manifests in root size and weight in radish [28], leaf weight in Chinese cabbage [29], and seed yield in oilseed rape [30]. However, for the allodiploid x*Brassicoraphanus* (Fig. S3), the plants produced smaller roots than radish and fewer leaves than Chinese cabbage. Thus, for different genera, the manifestation of heterosis is different, as previously indicated by East [11].

In the glass house, plant height or inflorescence stem length is another indicator of growth vigor of x*Brassicoraphanus*. Both *B. rapa* and *R. sativus* have an indeterminate flowering habitat; however, in the glass house, the growth of the parents was limited, with the length of inflorescence stem reaching approximately 1 m, in contrast to the hybrids with inflorescence stem length reaching over 2 m and showing more indeterminate growth in this habitat. Thus, the primary phenotype of heterosis was likely growth vigor expressed throughout all developmental stages; however, a different phenotype could occur in a different environment, as previously indicated [4, 31].

### Alteration in primary metabolism is likely related to heterosis

The DEGs and enriched GO terms in F1 hybrids are consistent with observations for hybrid rice [32] and Arabidopsis [27], in which photosynthesis-related genes are up-regulated and stress-response-related genes are down-regulated. These studies suggest that heterosis in F1 hybrid plants involves fundamental changes in energy production and response to environmental stimuli. In addition, these DEGs were specific to the parent of origin, i.e., the maternal copies of photosynthesis genes were up-regulated while the paternal copies were not. The altered sugar contents, differential expression of photosynthesis-related genes, and increased biomass in this study are consistent with the observations previously made in Arabidopsis, rice, and Chinese cabbage [27, 29, 31, 33].

Response to biotic and abiotic stresses is crucial for the adaptation to changing environments, which requires changes in metabolism and the production of diverse defense-related metabolites. Plant secondary metabolism involves many genes and the expenditure of much energy. Increased heterozygosity, particularly in an unstable intergeneric hybrid, may heighten the complexity of primary and secondary metabolism, and a finely tuned process is required for the production of secondary metabolites related to the response to stress. Our observations of heterosis in the F1 hybrids of x*Brassicoraphanus* led us to hypothesize that a balance between primary and secondary metabolism shifted in favor of primary metabolism, which was also accompanied by transcriptomic and metabolic changes, as exemplified by the alteration of both sugar and sugar acid concentrations and corresponding gene expression. Considering similar changes observed in Arabidopsis, rice, and other plants, our results suggest that a shift in sugar metabolism is essential to explain heterosis in hybrid plants [27, 32, 33].

### Existing models explain the heterosis only in part

Models of dominance, overdominance, and epitasis in gene expression have all been proposed by the transcriptome analysis of maize F1 hybrids [34]. However, allelic interactions that explain such models do not support the heterosis phenomenon observed in x*Brassicoraphanus* because all chromosomes exist in haploid configuration. Duplicate gene interactions are not necessarily allelic or under the control of Mendelian genetics [11]. Therefore, it is conceivable that heterosis in allodiploid hybrids is primarily regulated by the interplay between nonallelic genes rather than by allelic interactions. Thus the epistasis model which emphasizing inter-loci interaction would be more suitable than others in this case.

In recent models, energy use efficiency is used to explain heterosis, with hybrids reducing energy consumption during baseline metabolism and using the saved energy to increase biomass [7, 8]. The altered expression of metabolite-related genes (Fig. 5) and changes in metabolite profile (Fig. S3) may also contribute to the heterosis phenotypes observed in this study, although the link between altered gene expression and energy use efficiency needs further investigation. In addition, efficient sugar transport from source to sink is another issue for superior biomass in this case, since enlarged root or heading leaves are not present anymore in the hybrid, and absence of predominant sink could be attributed for the biomass heterosis. We focus on leaf metabolite and transcriptome in this study, however, different tissues such as root should be investigated for future analysis.

### Heterosis is important for evolution of novel species

Heterosis occurs in many organisms, and the role of heterosis in evolution has been discussed elsewhere [4, 35]. Superior phenotypes are often observed in interspecific hybrids and allopolyploids [11]. Considering that hybridization and polyploidization are regarded as important driving forces of evolution, our observations provide further evidence for an important role of heterosis in evolution to increase the fitness of the hybrid plants. With heterosis expressed in the hybrids, the possibility for newly hybridized organisms to survive may be dramatically increased by polyploidization, which should promote the genome stabilization that leads to acquisition of fertility and establishment of novel species.

Domestication or natural selection may lead to evolution in a direction in which primary and secondary metabolism are coordinated in adaptation to the environment rather than for simple growth. Plants likely developed mechanisms to control overgrowth because an optimal biomass is ecologically beneficial. However, with heterosis, plants may return to a less regulated condition that is more vigorous, leading to the expression of abilities that were lost during evolution (Fig. S9). Because growth vigor is lost within a few generations, hybrids apparently rapidly develop their adaptation metabolism.

The phenotypic variations observed in this study, such as flowering time and leaf shapes, suggested that instability existed in the F1 hybrid genome; however, we consistently observed heterosis in all hybrids, despite such variations. Based on this observation, it seems that the heterosis is not necessarily the result of allelic interactions, such as dominance and overdominance. By contrast, we hypothesized that when the two sets of genomes derived from discrete parents combined in a nucleus with maternal cytoplasm, the incompatibility caused the vigorous growth of the hybrid. Such a mechanism would also be associated with highly sophisticated regulation as an organism evolved. Thus, the heterosis that results from the incompatibility releases the high level of regulation and an organism is returned to a state of relatively unlimited growth (Fig. S9).

It remains to be determined which factors stimulate primary metabolism and repress secondary metabolism, returning the hybrid to the unrefined condition. One could assume that the regulatory genes for metabolism were the factors, which could be called heterosis genes. In addition, we propose that heterosis can result from any type of genetic or epigenetic disorders caused by the hybridization of distinct genomes that breaks the fine-tuned regulation.

## Conclusion

In this study, the biomass heterosis of F1 hybrids from an intergeneric cross between *B. rapa* and *R. sativus* was investigated with transcriptome and metabolite analysis. Consistent biomass heterosis despite of phenotypic variations among F1 hybrids, the differential expression of primary and secondary metabolism related genes, and the altered metabolite profiles suggest that heterosis could involve a change in balance between primary and secondary metabolism and corresponding transcriptome changes. The observations made in x*Brassicoraphanus*, a classic heterosis example, could provide different perspectives for understanding the underlying mechanism of heterosis.

## Methods

### Plant materials

The inbred lines of *B. rapa* cv. Chiifu and *R. sativus* cv. WK10039, used for the reference genome sequencing of *B. rapa* and *R. sativus* [21, 22], were kindly provided by Drs. Jin A Kim and Suhyung Park (Rural Development Administration, Wanju, Korea), respectively. The CF and WK plants were crossed to produce F1 hybrids (Figs. 1, 2, and S1). Unopened CF buds were emasculated and hand-pollinated with matured WK pollen. Two weeks after pollination, siliques were harvested and sterilized in a 50% bleach solution for 15 min with continuous inversion, followed by rinsing three times with distilled water. The immature seeds were removed from the siliques on a clean bench and cultured on MS medium until the plantlets were generated. The plantlets on the MS medium were transferred to soil (Sunshine Mix #5, SunGro, USA) and grown in a walk-in chamber at 24 °C under a 16-h day for a month. The plantlets were then transplanted to a bigger pot in the glass house or to the field. The CF and WK seeds were also placed on the MS medium and transferred to soil at the similar developmental stage, which was approximately 1 week. Parental plants were grown for a month in a walk-in chamber together with F1 plants and then transferred either in pots in a glass house or in the field. For plants grown in the glass house, flowering time, stem length, and node number were measured. Flowering time was determined by the days after planting when the bolting is first visible. Stem length and node number were counted from 3 to 5 fully developed inflorescence stems for each plant and at least three plant of parents and F1 hybrids as indicated in Table 1. For plants grown in the field, plant fresh weight and leaf number were measured from more than three plants for parents and F1 as indicated in Table 1.

### RNA sequencing

Plants transplanted from in vitro immature seed rescue and grown a month in pots in a walk-in chamber were sampled for RNA sequencing. To minimize the effect of a growth difference between the F1 and parent plants, the samples for each plant were a mixture of three leaves at different stages (young, middle, and fully developed) of development; these leaves were photographed (Fig. S1) and then frozen and ground together in liquid nitrogen. Total RNA was extracted with a Plant RNeasy mini kit (Qiagen, Germany) following on-column DNase treatment according to the manufacturer’s instructions. A Truseq RNA library was constructed, as reported previously [36], and sequenced on a HiSeq 2000 platform (Illumina, USA).

### Quantitative PCR analysis

To validate RNA sequencing results, selected orthologous pair genes were used for qPCR analysis with three biological replicates of CF, WK, and F1. Total RNA (1 μg) was used for reverse transcription with Superscript III Reverse transcriptase (Invitrogen, USA) and 1 μL of 1/20 diluted cDNA was used for a PCR reaction. Primers were listed in Table S5. qPCR was performed for 10 min of denaturation at 95 °C following 40 cycles of 10 s at 95 °C, 15 s at 60 °C, and 35 s at 72 °C with Rotor-Gene Q 2plex HRM platform and QuantiFast SYBR Green PCR kit (Qiagen, Germany).

### Analysis of transcriptome data

The low-quality raw reads (<Q20) were filtered with the CLC-quality trim tool, and the duplicated reads were removed using fastUniq 1.0 [37]. The filtered reads were mapped to the genomes of *B. rapa* (Brassica_rapa.IVFCAASv1.31, https://plants.ensembl.org/Brassica_rapa) and *R. sativus* (Rs_1.0, http://radish-genome.org/) using TopHat v2.0.13 [38] with default parameters. The mapped read counts were calculated using HTSeq 0.6.1p1 [39]. After calling the read counts, the EdgeR Bioconductor package version 3.8.6 [40] was used for the statistical analysis of DEGs. DEGs were defined as genes with a false discovery rate (FDR) less than 0.05, an over 2-fold change in fragments per kilobase million (FPKM), and a minimum expression of 0.3 FPKM. The orthologous genes between *B. rapa* (A) and *R. sativus* (R) genomes were found using BLAST with the reciprocal best hit. For the Gene Ontology (GO) analysis, Blast2GO [41] was used to annotate the GO terms using the NCBI nr database, and the topGO R package version 2.18.0 was used to perform the GO term enrichment test [42]. For the KEGG pathway analysis, the KEGG automatic annotation server was used to annotate the terms [43], and the enrichment tests were performed using the KEGG database (http://www.genome.jp/kegg/).

### Metabolite analysis

The polar metabolites were extracted with methanol, as described in a previous study with some modifications [44]. Freeze-dried leaf samples of CF, WK, and CWB were powdered with a coffee grinder. Each 50-mg subsample of powder was mixed with 1.2 mL of methanol and vortexed vigorously. An autosampler injected 1 μL of methanol extracts into a GC-MS ISQLT system (Thermo Fisher Scientific, USA). The samples included four biological replicates for CF and WK and 19 for CWB (Table S1). The polar metabolite data were processed for normalization and principal component analysis (PCA) using MetaboAnalyst 3.0 software (www.metaboanalyst.ca).

## Supplementary information

**Additional file 1: Table S1.** Summary of F1 samples and their usage for analysis (DOCX 2531 kb). **Table S2.** Summary of RNA-seq reads. **Table S3.** Top 5 enriched GO terms of the genes having expression correlation with the phenotypes in F1 hybrids. **Table S4.** Top 5 enriched GO terms of the genes have expression correlation with the metabolite contents in F1 hybrids. **Table S5.** Orthologous pair genes and their primers used for qRT-PCR analysis. **Figure S1.** Leaf shapes of the newly synthesized allodiploid x*Brassicoraphanus*. **Figure S2.** Pollen shapes of the newly synthesized allodiploid x*Brassicoraphanus*. **Figure S3.** Chromosome numbers for CF, WK, and CWB. 19 chromosomes (univalents) were observed in allodiploid F1 hybrid (C), whereas 10 and 9 chromosome pairs (bivalents) were observed in CF (A) and WK (B), respectively. Chromosome spreads were obtained from pollen mother cells in diakinesis of meiosis. Bars = 10 μm. **Figure S4.** Concentrations of metabolites for four biological replicates for the parents (CF and WK) and 19 biological replicates for the F1 hybrids (F1) as indicated on the top of each graph. **Figure S5.** Validation of RNA-seq results by qPCR. **Figure S6.** Validation of RNA-seq results by qPCR by species specific and general primers. **Figure S7.** Assignment of DEGs in metabolism-related KEGG pathways. **Figure S8.** Correlations between the phenotypes and the metabolite concentrations and among the metabolite concentrations. **Figure S9.** Diagram showing the proposed mechanism of heterosis.

## Abbreviations

CF: *B. rapa* cv. Chiifu
CWB: F1 hybrids from a cross between *B. rapa* cv. Chiifu and *R. sativus* cv. WK10039
DEG: Differentially expressed gene
FDR: False discovery rate
FPKM: Fragments per kilobase million
GO: Gene ontology
KEGG: Kyoto encyclopedia of genes and genomes
MPV: Middle parent value
PCA: Principal component analysis
WK: *R. sativus* cv. WK10039
