# Supplementary material for: Revealing biomass heterosis in the allodiploid xBrassicoraphanus, a hybrid between Brassica rapa and Raphanus sativus, through integrated transcriptome and metabolites analysis
Source: BMC Plant Biol. 2020 Jun 3;20:252. doi: 10.1186/s12870-020-02470-9 (PMC7268423; doi:10.1186/s12870-020-02470-9)
Supplement: Supplementary file 1 — Additional file 1: Table S1. Summary of F1 samples and their usage for analysis (DOCX 2531 kb). Table S2. Summary of RNA-seq reads. Table S3. Top 5 enriched GO terms of the genes having expression correlation with the phenotypes in F1 hybrids. Table S4. Top 5 enriched GO terms of the genes have expression correlation with the metabolite contents in F1 hybrids. Table S5. Orthologous pair genes and their primers used for qRT-PCR analysis. Figure S1. Leaf shapes of the newly synthesized allodiploid xBrassicoraphanus. Figure S2. Pollen shapes of the newly synthesized allodiploid xBrassicoraphanus. Figure S3. Chromosome numbers for CF, WK, and CWB. 19 chromosomes (univalents) were observed in allodiploid F1 hybrid (C), whereas 10 and 9 chromosome pairs (bivalents) were observed in CF (A) and WK (B), respectively. Chromosome spreads were obtained from pollen mother cells in diakinesis of meiosis. Bars = 10 μm. Figure S4. Concentrations of metabolites for four biological replicates for the parents (CF and WK) and 19 biological replicates for the F1 hybrids (F1) as indicated on the top of each graph. Figure S5. Validation of RNA-seq results by qPCR. Figure S6. Validation of RNA-seq results by qPCR by species specific and general primers. Figure S7. Assignment of DEGs in metabolism-related KEGG pathways. Figure S8. Correlations between the phenotypes and the metabolite concentrations and among the metabolite concentrations. Figure S9. Diagram showing the proposed mechanism of heterosis. [file 12870_2020_2470_MOESM1_ESM.docx]

**Revealing biomass heterosis in the allodiploid x*Brassicoraphanus*, a hybrid between *Brassica rapa* and *Raphanus sativus*, through integrated transcriptome and metabolites analysis**

Gibum Yi^1,2*†^, Hosub Shin^1^, Hye Rang Park^1^, Jeong Eun Park^1^, Jong Hwa Ahn^1#^, Sooyeon Lim^1,3‡^, Jeong Gu Lee^1^, Eun Jin Lee^1,3^, and Jin Hoe Huh^1,2,3*^

**Supplementary Information**

This Supplementary Information contains 5 Supplementary Tables and 9 Supplementary Figures

**Supplementary Table S1**. Summary of F1 samples and their usage for analysis

| # | ID | Growth condition | Leaf shape | RNA-seq | Metabolites analysis | Flowering time | Stem length & node number | Biomass in field |
| --- | --- | --- | --- | --- | --- | --- | --- | --- |
| 1 | CWB1 | A glass house | o | x | x | o | o | x |
| 2 | CWB2 | " | o | o | o | o | o | x |
| 3 | CWB3 | " | o | o | o | o | x | x |
| 4 | CWB4 | " | o | o | o | o | x | x |
| 5 | CWB5 | " | o | o | o | o | o | x |
| 6 | CWB6 | " | o | o | o | o | x | x |
| 7 | CWB7 | " | o | x | x | o | o | x |
| 8 | CWB8 | " | o | o | o | o | o | x |
| 9 | CWB9 | " | o | o | o | o | o | x |
| 10 | CWB10 | " | o | o | o | o | o | x |
| 11 | CWB13 | " | o | o | o | o | o | x |
| 12 | CWB14 | " | o | o | o | o | o | x |
| 13 | CWB15 | " | o | x | x | o | x | x |
| 14 | CWB16 | " | o | o | o | o | o | x |
| 15 | CWB17 | " | o | o | o | o | o | x |
| 16 | CWB18 | " | o | o | o | o | o | x |
| 17 | CWB19 | " | o | o | o | o | o | x |
| 18 | CWB20 | " | o | o | o | o | o | x |
| 19 | CWB21 | " | o | o | o | o | o | x |
| 20 | CWB22 | " | o | o | x | o | o | x |
| 21 | CWB23 | " | o | o | o | o | o | x |
| 22 | CWB24 | " | o | o | o | o | o | x |
| 23 | CWB25 | " | o | x | x | o | o | x |
| 24 | CWB26 | " | o | x | x | o | o | x |
| 25 | CWB27 | " | o | o | o | o | o | x |
| 26 | CWB28 | " | o | x | x | o | o | x |
| 27 | CWB29 | " | o | x | x | o | o | x |
| 28 | CWB30 | " | o | x | x | o | o | x |
| 29 | CWB101 | field | x | x | x | x | x | o |
| 30 | CWB102 | field | x | x | x | x | x | o |
| 31 | CWB103 | field | x | x | x | x | x | o |
| Total number | |  | 28 | 20 | 19 | 28 | 24 | 3 |

#: number, ": same as above, o: used, x: not used.

**Supplementary Table S2**. Summary of RNA-seq reads

|  | Sample Name | Total read length (bp) | Total reads No. | GC(%) | Q20(%) |
| --- | --- | --- | --- | --- | --- |
| 1 | CF1 | 1,636,965,378 | 16,207,578 | 48.18 | 95.25 |
| 2 | CF2 | 1,661,218,912 | 16,447,712 | 48.46 | 95.40 |
| 3 | CF3 | 1,643,272,222 | 16,270,022 | 49.25 | 95.48 |
| 4 | WK1 | 1,663,512,420 | 16,470,420 | 48.56 | 94.87 |
| 5 | WK2 | 1,966,988,332 | 19,475,132 | 48.42 | 95.15 |
| 6 | WK3 | 1,748,116,686 | 17,308,086 | 49.49 | 95.19 |
| 7 | CWB2 | 1,784,044,002 | 17,663,802 | 48.07 | 95.16 |
| 8 | CWB3 | 1,539,276,966 | 15,240,366 | 48.29 | 94.99 |
| 9 | CWB4 | 1,530,390,784 | 15,152,384 | 48.45 | 95.07 |
| 10 | CWB5 | 1,708,073,620 | 16,911,620 | 48.73 | 95.35 |
| 11 | CWB6 | 1,794,083,402 | 17,763,202 | 48.12 | 95.19 |
| 12 | CWB8 | 1,367,639,384 | 13,540,984 | 48.25 | 95.37 |
| 13 | CWB9 | 1,908,672,548 | 18,897,748 | 48.21 | 95.52 |
| 14 | CWB10 | 1,441,299,290 | 14,270,290 | 48.67 | 95.26 |
| 15 | CWB13 | 1,441,838,024 | 14,275,624 | 48.90 | 95.27 |
| 16 | CWB14 | 2,775,471,516 | 27,479,916 | 48.55 | 95.36 |
| 17 | CWB16 | 1,406,150,886 | 13,922,286 | 48.67 | 95.20 |
| 18 | CWB17 | 2,604,284,394 | 25,784,994 | 48.25 | 95.90 |
| 19 | CWB18 | 1,361,269,718 | 13,477,918 | 48.61 | 95.35 |
| 20 | CWB19 | 1,641,805,500 | 16,255,500 | 48.63 | 95.39 |
| 21 | CWB20 | 1,788,644,350 | 17,709,350 | 48.36 | 95.45 |
| 22 | CWB21 | 1,541,955,486 | 15,266,886 | 48.40 | 95.24 |
| 23 | CWB22 | 1,218,606,208 | 12,065,408 | 48.89 | 95.41 |
| 24 | CWB23 | 1,347,855,100 | 13,345,100 | 49.22 | 95.31 |
| 25 | CWB24 | 1,440,267,676 | 14,260,076 | 48.93 | 95.10 |
| 26 | CWB27 | 1,630,186,258 | 16,140,458 | 49.08 | 95.28 |

**Supplementary Table S3**. Top 5 enriched GO terms of the genes having expression correlation with the phenotypes in F1 hybrids

| Correlation | GO | Term | Annotated | Significant | Expected | Fisher  (<0.001) | FDR | Level |
| --- | --- | --- | --- | --- | --- | --- | --- | --- |
| Floral stem length | GO:0006633 | fatty acid biosynthetic process | 564 | 16 | 5.81 | 0.00029 | 0.908 | 6 |
|  | GO:0072330 | monocarboxylic acid biosynthetic process | 842 | 20 | 8.67 | 0.00055 | 0.908 | 7 |
|  | GO:0016311 | dephosphorylation | 729 | 18 | 7.51 | 0.00066 | 0.908 | 6 |
|  | GO:0035435 | phosphate ion transmembrane transport | 17 | 3 | 0.18 | 0.00066 | 0.908 | 6 |
| Floral node number | GO:0043436 | oxoacid metabolic process | 3681 | 89 | 40.93 | 2.70E-12 | 8.26E-09 | 5 |
|  | GO:0006082 | organic acid metabolic process | 3688 | 89 | 41.01 | 3.00E-12 | 8.26E-09 | 4 |
|  | GO:0006568 | tryptophan metabolic process | 72 | 12 | 0.8 | 2.60E-11 | 3.58E-08 | 6 |
|  | GO:0006586 | indolalkylamine metabolic process | 72 | 12 | 0.8 | 2.60E-11 | 3.58E-08 | 6 |
|  | GO:0009611 | response to wounding | 758 | 32 | 8.43 | 1.80E-10 | 1.98E-07 | 4 |
| Flowering time | GO:0030244 | cellulose biosynthetic process | 171 | 23 | 2.63 | 3.10E-15 | 1.71E-11 | 8 |
|  | GO:0051274 | beta-glucan biosynthetic process | 209 | 24 | 3.21 | 2.80E-14 | 7.71E-11 | 7 |
|  | GO:0030243 | cellulose metabolic process | 251 | 24 | 3.86 | 1.60E-12 | 2.94E-09 | 8 |
|  | GO:0051273 | beta-glucan metabolic process | 289 | 25 | 4.44 | 5.00E-12 | 6.49E-09 | 7 |
|  | GO:0034637 | cellular carbohydrate biosynthetic process | 599 | 36 | 9.21 | 5.90E-12 | 6.49E-09 | 4 |

| Correlation | GO | Term | Annotated | Significant | Expected | Fisher(<0.001) | FDR | level |
| --- | --- | --- | --- | --- | --- | --- | --- | --- |
| D-gluconic acid | GO:0019676 | ammonia assimilation cycle | 22 | 4 | 0.26 | 0.00011 | 0.358 | 8 |
|  | GO:0080114 | positive regulation of glycine hydroxymethyltransferase activity | 2 | 2 | 0.02 | 0.00013 | 0.358 | 5 |
|  | GO:0006892 | post-Golgi vesicle-mediated transport | 49 | 5 | 0.57 | 0.00026 | 0.385 | 6 |
|  | GO:0034337 | RNA folding | 3 | 2 | 0.03 | 0.0004 | 0.385 | 6 |
|  | GO:0009657 | plastid organization | 578 | 17 | 6.71 | 0.0005 | 0.385 | 4 |
| succinic acid | none |  |  |  |  |  |  |  |
| mannose | GO:0009833 | primary cell wall biogenesis | 26 | 5 | 0.34 | 2.00E-05 | 0.110 | 6 |
|  | GO:0010563 | negative regulation of phosphorus metabolic process | 39 | 5 | 0.51 | 0.00015 | 0.275 | 5 |
|  | GO:0045936 | negative regulation of phosphate metabolic process | 39 | 5 | 0.51 | 0.00015 | 0.275 | 6 |
|  | GO:0007568 | aging | 546 | 18 | 7.17 | 0.00039 | 0.351 | 4 |
|  | GO:0002119 | nematode larval development | 3 | 2 | 0.04 | 0.00051 | 0.351 | 6 |
| D-fructose | GO:0009833 | primary cell wall biogenesis | 26 | 5 | 0.26 | 5.60E-06 | 0.031 | 6 |
|  | GO:0002119 | nematode larval development | 3 | 2 | 0.03 | 0.0003 | 0.205 | 6 |
|  | GO:0002164 | larval development | 3 | 2 | 0.03 | 0.0003 | 0.205 | 5 |
|  | GO:0034337 | RNA folding | 3 | 2 | 0.03 | 0.0003 | 0.205 | 6 |
|  | GO:0061062 | regulation of nematode larval development | 3 | 2 | 0.03 | 0.0003 | 0.205 | 6 |
| O-glycerol-a-d-galactopyranoside | GO:0070141 | response to UV-A | 11 | 6 | 0.18 | 7.10E-09 | 1.30E-05 | 7 |
|  | GO:0071492 | cellular response to UV-A | 11 | 6 | 0.18 | 7.10E-09 | 1.30E-05 | 8 |
|  | GO:0071486 | cellular response to high light intensity | 18 | 7 | 0.29 | 7.10E-09 | 1.30E-05 | 8 |
|  | GO:0034644 | cellular response to UV | 28 | 8 | 0.45 | 9.60E-09 | 1.32E-05 | 7 |
|  | GO:0071484 | cellular response to light intensity | 26 | 7 | 0.42 | 1.30E-07 | 0.00014 | 7 |

**Supplementary Table S4**. Top 5 enriched GO terms of the genes have expression correlation with the metabolite contents in F1 hybrids

**Supplementary Table S5**. Orthologous pair genes and their primers used for qRT-PCR analysis

| Orthologous pair | | Primer name | Sequence | Gene description |
| --- | --- | --- | --- | --- |
| Bra022643 | Rs070100 | BrMSI1-F | aggatttggctcggtactttc | *MSI1* (multicopy supressor of IRA1) |
|  |  | BrMSI1-R | tgctccggttctgattcatc |  |
| Bra015815 | Rs387530 | BrTUB1-F | ctctcaaactcagcactcctag | *TUB1*; GTP binding |
|  |  | BrTUB1-R | ttcacggcgagtttccttag |  |
| Bra021505 | Rs295510 | BrCYP90D1-F | tttggccgtcttgaagct | *CYP90D1*; oxidoreductase |
|  |  | BrCYP90D1-R | tcatatgcactgtaggaaaatttataattg |  |
| Bra007924 | Rs070310 | BrWEI8-F | cccatcactcgctgtcaag | *WEI8*, *TAA1* (tryptophan aminotransferase) |
|  |  | BrWEI8-R | tccttgtccttcaccaatgc |  |
| Bra013344 | Rs051360 | BrDOT1-F | agagaagaagggtactcgccgca | *DOT1* (defectively organized tributaries 1) |
|  |  | BrDOT1-R | gtccagaggtgagtatccccaacc |  |
|  |  | RsDOT1-F | agagaagaagggtactcgccgct |  |
|  |  | RsDOT1-R | gtccagaggttagtatccccaacg |  |
|  |  | BRsDOT1-F | agagaagaagggtactcgccgc |  |
|  |  | BRsDOT1-R | catgtccagaggtgagtatccccaac |  |
| Bra040094 | Rs060970 | BRsHB-2-F | acggagctgaggtcgcttaagc | *ATHB-2* (arabidopsis thaliana homeobox protein 2) |
|  |  | BrHB-2-R | tccggacgcagcaacttgaggtg |  |
|  |  | RsHB2-R2 | gcatcgacctctggtggtgtccta |  |
|  |  | BRsHB2-F2 | tctgctattctcgaagagaccttc |  |
|  |  | BRsHB2-R2 | ccttgctcgtctgttctgaaacc |  |
| Bra009474 | Rs389820 | BrELF6-F | tgtgaagggttgaggtcaag | *ELF6* (early flowering 6) |
|  |  | BrELF6-R | gttgtcgttgagccagtatttg |  |
|  |  | RsELF6-F2 | tgtgaagggttgaggtcaaggggt |  |
|  |  | RsELF6-R2 | cttggccatgagtcctcgtgcg |  |
|  |  | BRsELF6-F2 | gcaatgggcgaggactgagc |  |
|  |  | BRsELF6-R2 | ccgtctgtggcggctataatccg |  |
| Bra011955 | Rs353080 | BrARF10-F | ccgatttgttgactcatgttgtg | *ARF10* (auxin response factor 10) |
|  |  | BrARF10-R | ctttctcacgttgtcgccgctt |  |
|  |  | RsARF10-F | cggatttgttgacccatgttgtt |  |
|  |  | RsARF10-R | ctttctcaagttgtcgccgcca |  |
|  |  | BRsARF10-F2 | atttcatgagagcaacgaagcgact |  |
|  |  | BRsARF10-R2 | tcaagcgaagatgctaagctgaccg |  |


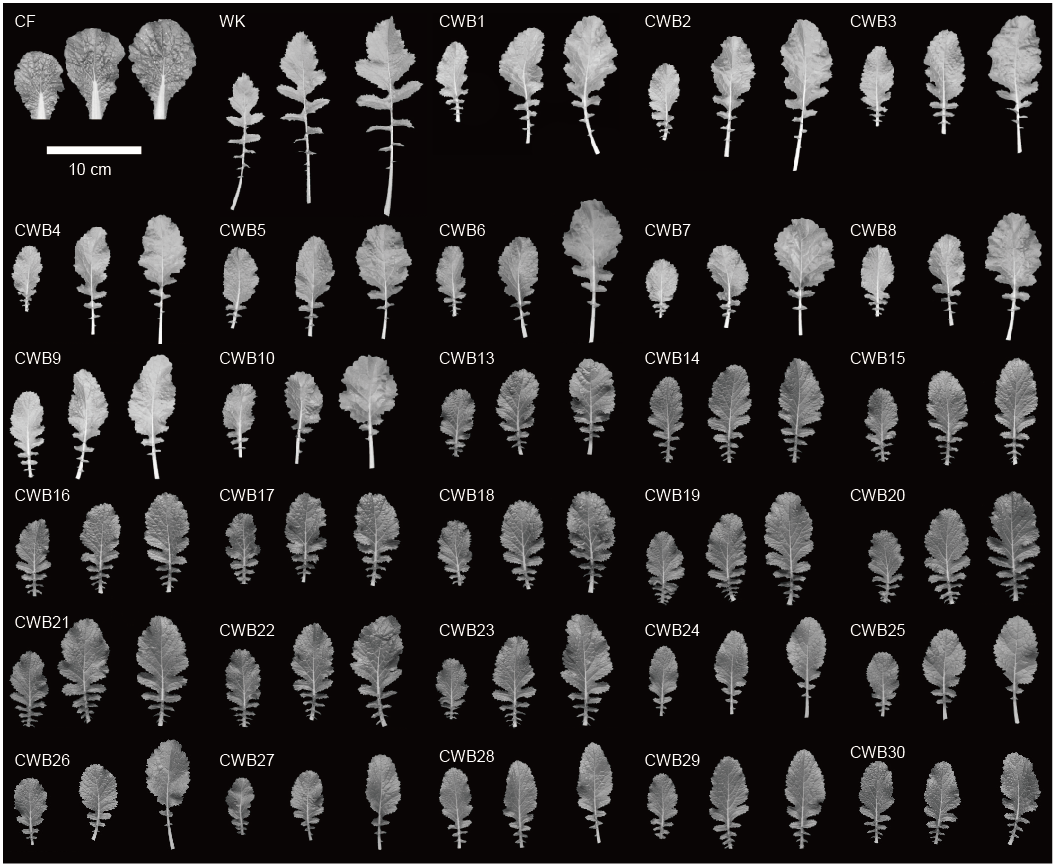


**Supplementary Fig. S1**. Leaf shapes of the newly synthesized allodiploid x*Brassicoraphanus*. Plants were recovered from ovules and grown in vitro for a month and transplanted in soil in 10-cm-diameter pots. When plants were grown a month in the pot, three leaves from different developmental stages are pictured together. Separate pictures were combined with the same scale; bar = 10 cm.


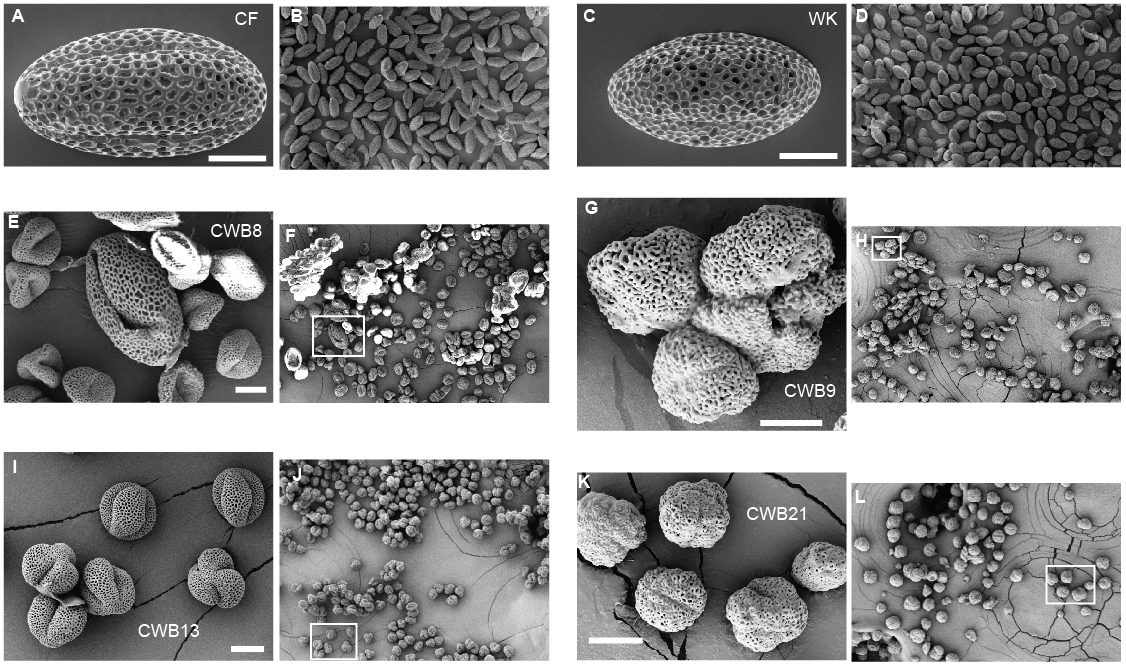


**Supplementary Fig. S2**. Pollen shapes of the newly synthesized allodiploid x*Brassicoraphanus*. (a, b) CF; (b, c) WK; (e, f) CWB8; (g, h) CWB9; (i, j) CWB13; (k, l) CWB21. Boxed regions in (f, h, j, and l) are magnified in (e, g, i, and k), respectively. Bars = 10 μm.


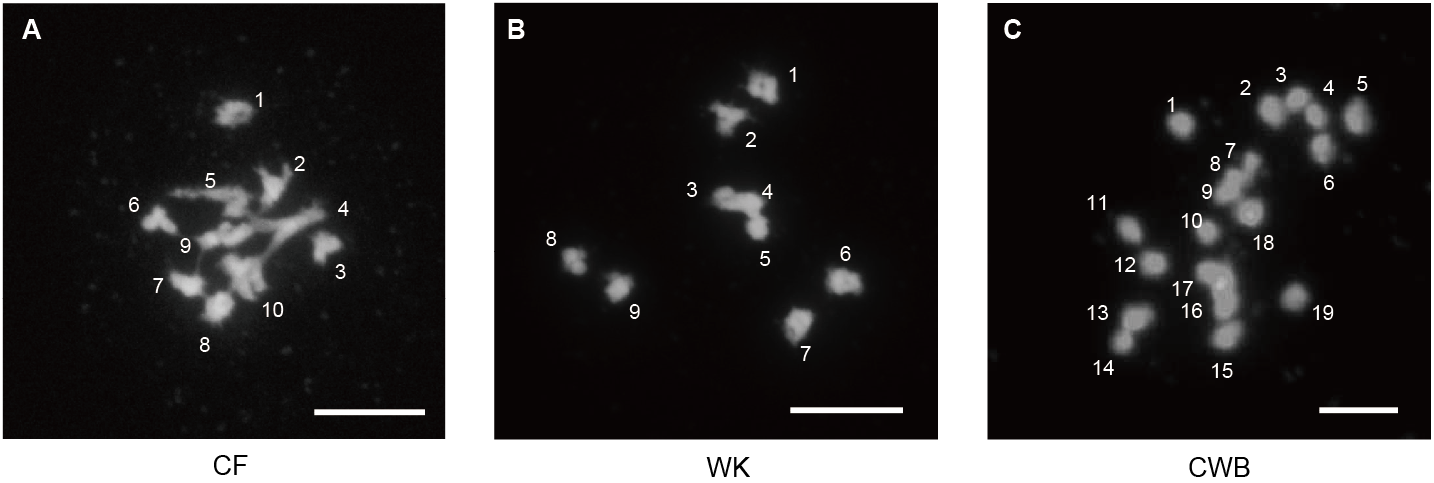


**Supplementary Fig. S3**. Chromosome numbers for CF, WK, and CWB. 19 chromosomes (univalents) were observed in allodiploid F1 hybrid (C), whereas 10 and 9 chromosome pairs (bivalents) were observed in CF (A) and WK (B), respectively. Chromosome spreads were obtained from pollen mother cells in diakinesis of meiosis. Bars = 10 μm


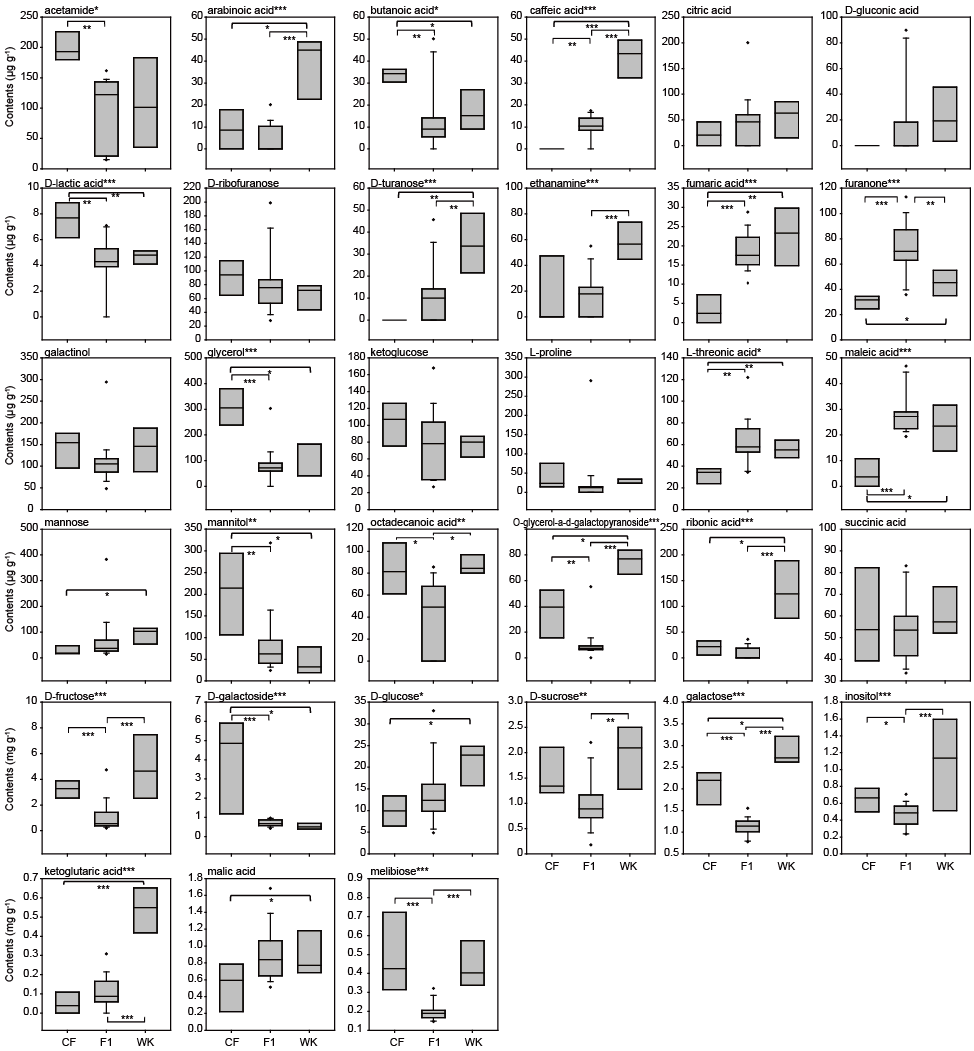


**Supplementary Fig. S4**. Concentrations of metabolites for four biological replicates for the parents (CW and WK) and 19 biological replicates for the F1 hybrids (F1) as indicated on the top of each graph. Differences among the F1 and parents were tested by ANOVA for each metabolite, and significance is indicated on the name of each metabolite as *p<0.05, **p<0.01, and ***p<0.001. Differences between samples are indicated in each graph by Student’s t-test as *p<0.05, **p<0.01, and ***p<0.001. Designations for *x*- and *y*-axis can be found on the bottom and on the left side, respectively.


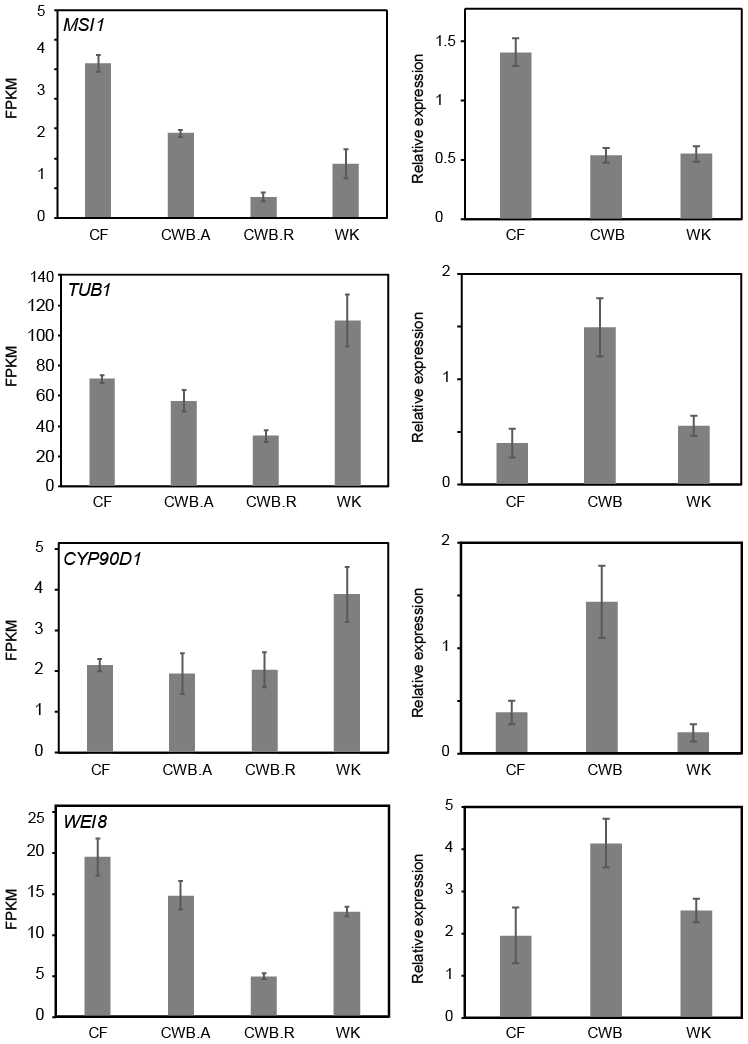


**Supplementary Fig. S5**. Validation of RNA-seq results by qPCR. Same amount of total RNA (1 μg) was used for RT-PCR for comparison of amount genes for *MSI1*, *TUB1*, *CYP90D1*, and *WEI8* as designated. The left and right column graphs are showing RNA-seq and qPCR result, respectively. Error bars represent standard deviations from biological replicates.


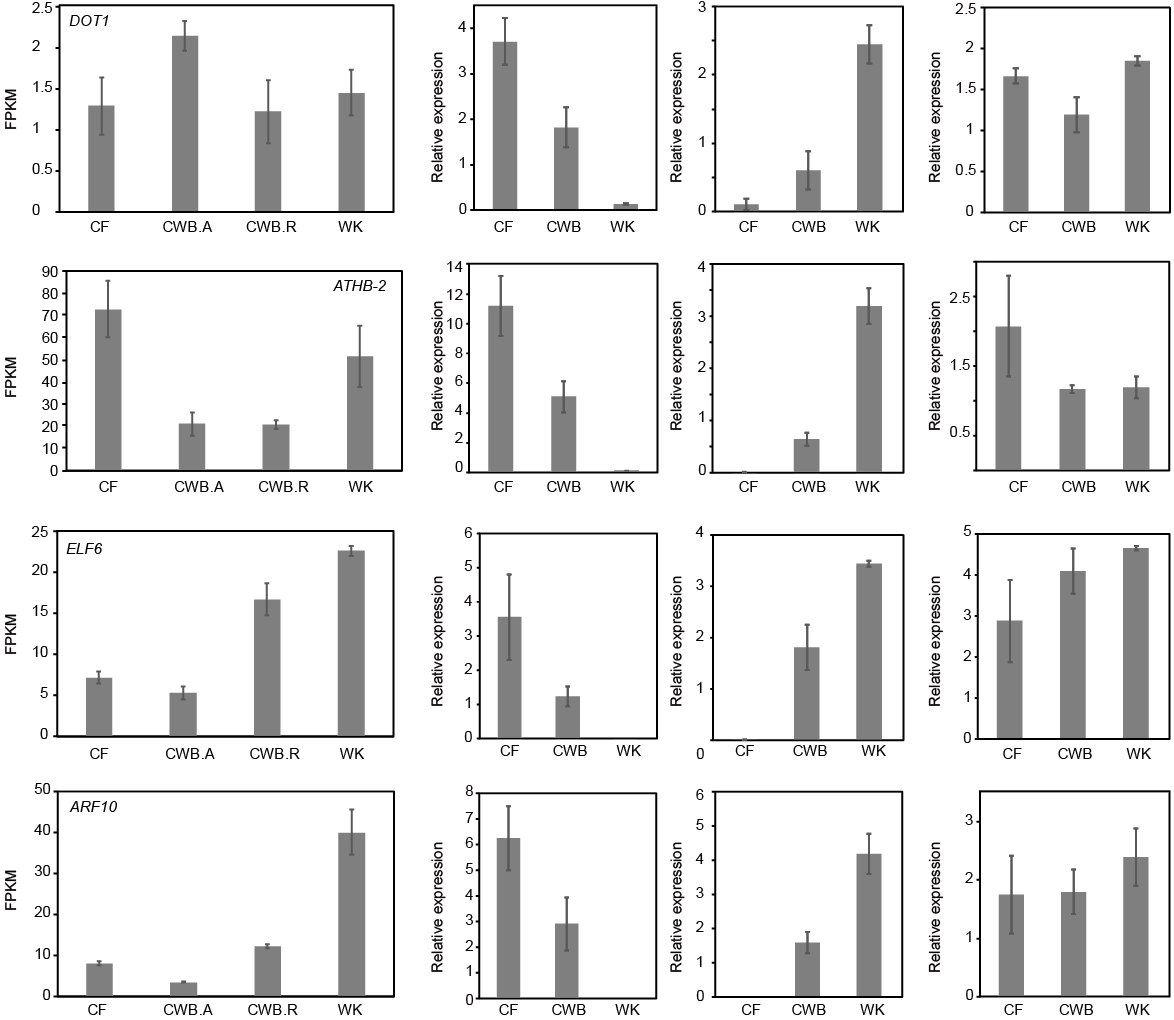


**Supplementary Fig. S6**. Validation of RNA-seq results by qPCR by species specific and general primers. *DOT1*, *ATHB-2*, *ELF6*, and *ARF10* gene primers were designed based on 5’ end mismatch for species specific amplification by comparing orthologous gene sequences. RNA-seq results were shown on the left column and the three right graphs are showing qPCR results with *B. rapa*-specific, *R. sativus*-specific, and general primers, respectively. Error bars represent standard deviations from biological replicates.


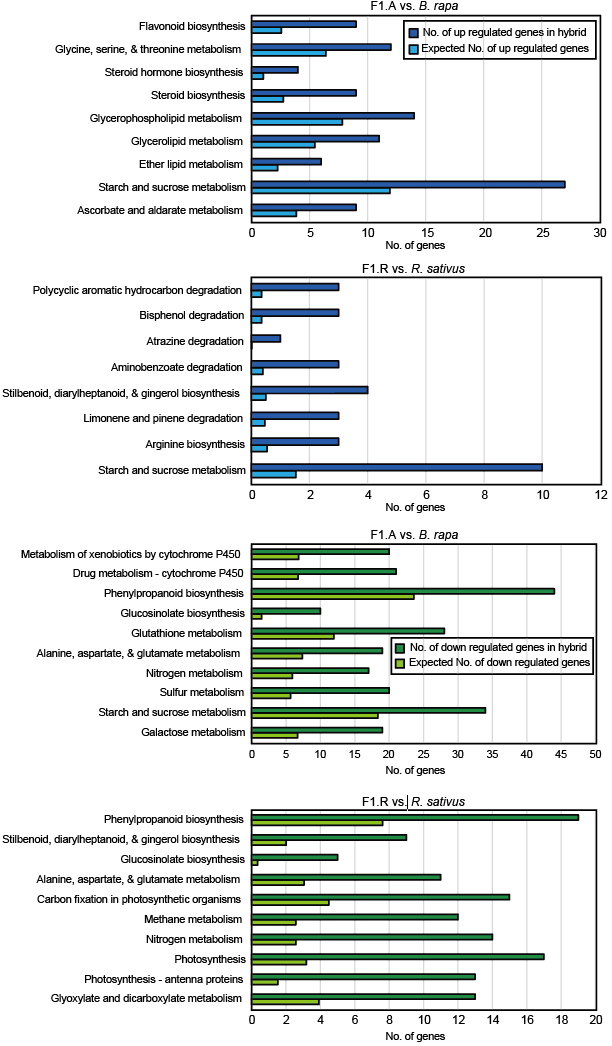


**Supplementary Fig. S7**. Assignment of DEGs in metabolism-related KEGG pathways. Enriched KEGG pathway genes with false discovery rate (p<0.05) are presented. Green and blue bars indicate up- and down-regulated genes in the hybrid, respectively. The *x*-axis is the number of corresponding genes.


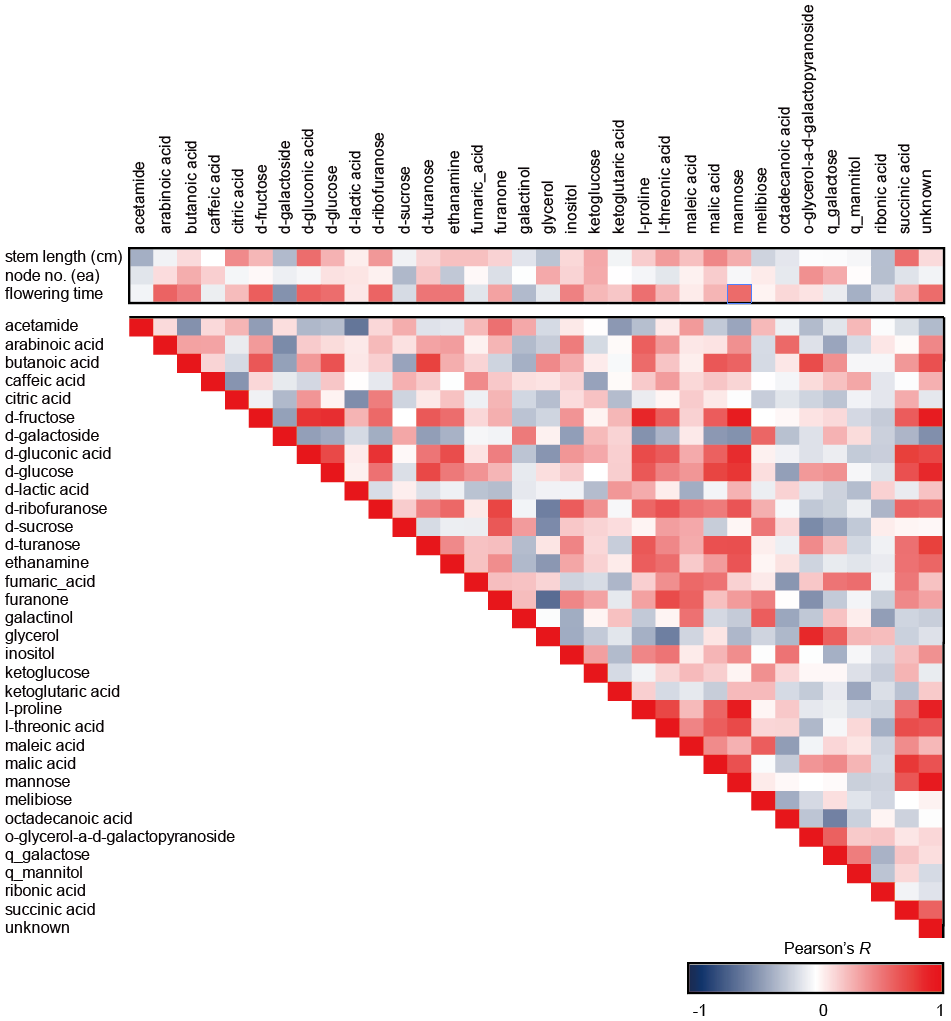


**Supplementary Fig. S8**. Correlations between the phenotypes and the metabolite concentrations and among the metabolite concentrations. Positive and negative correlations were visualized by Pearson’s R values indicated on the bottom.


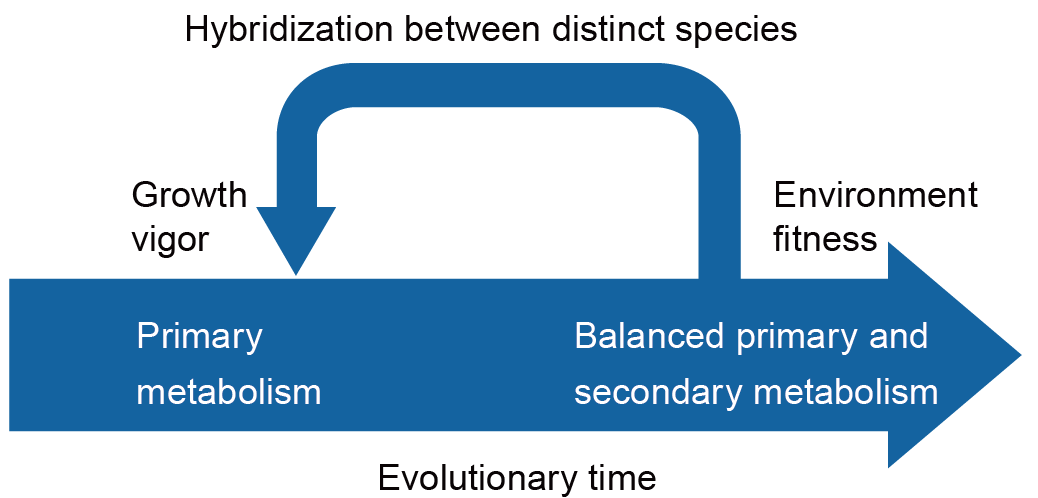


**Supplementary Fig. S9**. Diagram showing the proposed mechanism of heterosis.
